# A multi-zoned white organic light-emitting diode with high CRI and low color temperature

**DOI:** 10.1038/srep20517

**Published:** 2016-02-04

**Authors:** Tao Zhang, Shou-Jie He, Deng-Ke Wang, Nan Jiang, Zheng-Hong Lu

**Affiliations:** 1 Department of Physics, Yunnan University, Kunming, Yunnan 650091, People’s Republic of China; 2 Yunnan Key Laboratory for Micro/Nano Materials & Technology, Yunnan University, Kunming, Yunnan 650091, People’s Republic of China; 3 Department of Materials Science and Engineering, University of Toronto, Toronto, Ontario M5S 3E4, Canada

## Abstract

White organic light emitting diodes (WOLEDs) is becoming a new platform technology for a range of applications such as flat-panel displays, solid-state lightings etc., and are under intensive research. For general solid-state illumination applications, a WOLED’s color rendering index (CRI) and correlated color temperature (CCT) are two crucial parameters. This paper reports that WOLED device structures can be constructed using four stacked emission layers which independently emit lights at blue, green, yellow and red color respectively. The intensity of each emission layer is then engineered by funneling excitons to the targeted emission layer to achieve an ultrahigh 92 CRI at 5000 cd/m^2^, and to reduce CCT to below 2500 K.

Organic light emitting diodes (OLEDs) have received a broad attention for their enormous applications in the field of flat-panel displays and solid-state lightings^1^,^2^,^3^,^4^. For lighting applications, three light output characteristics are crucial: (i) the CIE (Commission Internationale d’Eclairage) coordinates (the chromaticity), (ii) the color temperature (CT), and (iii) the color rendering index (CRI)^5^. A light source’s CT is measured in reference to the blackbody radiation at a given temperature measured in Kelvin. Correlated color temperature (CCT) is the color appearance of a light source, measured by the source’s chromaticity in reference to the blackbody locus. CCT affects visual and emotional aspects of people under illumination. A lower CCT light source, which creates an atmosphere known to stimulate relaxation, is much desired in a multitude of locations^6^. Another critical aspect in a lighting system is the CRI, which is a measurement of how accurate the colors of objects can be rendered under a given illumination condition. The higher the CRI value of a light source the more accurate color it can render an illuminated object. Thus ultrahigh CRI lightings for demanding environment such as surgery, photography, exhibition in museums, printing industry etc. are extremely crucial^7^. Recently, there are several articles about high CRI WOLEDs^7^,^8^,^9^,^10^,^11^, for example, Jou *et al*. reported a WOLED with CRI of 96 and power efficiency (PE) of 5.2 lm/W at 1000 cd/m^2^ by incorporating double white emissive-layers. Ma *et al*. reported a green Plumbum (II) emitter device with CRI of 97 and PE of 0.6 lm/W at 15000 cd/m^2^. Yang *et al*. reported a triple emissive-layer (EML) device with CRI of 91 and PE of 3.1 lm/W. Leo *et al*. reported a device structure using a deep-blue fluorescent mixed with a green and a red phosphorescent dopants to generate a WOLED with CRI of 86 and PE of 22 lm/W at 1000 cd/m^2^. Chang *et al*. reported a four color dyes device with CRI of 84 and PE of 31 lm/W at 1000 cd/m^2^. Many published reports on high CRI WOLEDs show that there is still considerable room for improving the efficiency, CCT and CRI of devices. When placed in practical use, lamps should be bright for clear visibility for general applications, whereas for certain special applications the lamps are required to produce a relaxing and pleasant atmosphere. Therefore, the challenge for WOLED is to design and fabricate a high performance device with an ultrahigh CRI along with a tunable CCT character, especially in a low below 3000 K CCT range for the benefit of safeguarding human health.

In this paper, a four emission zone WOLED is demonstrated to be capable of achieving an ultrahigh CRI > 92 at high 5000 cd/m^2^ luminance with an extremely low CCT ~ 2500 K, while maintaining a high efficiency of ~25 lm/W without optical coupling.

## Results

**Figure 1:**
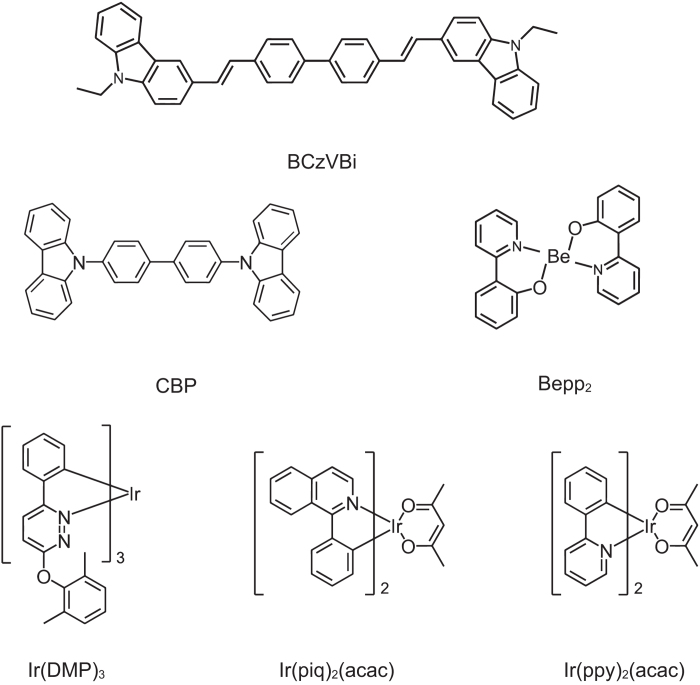
The chemical structures of the organic materials.

**Figure 2:**
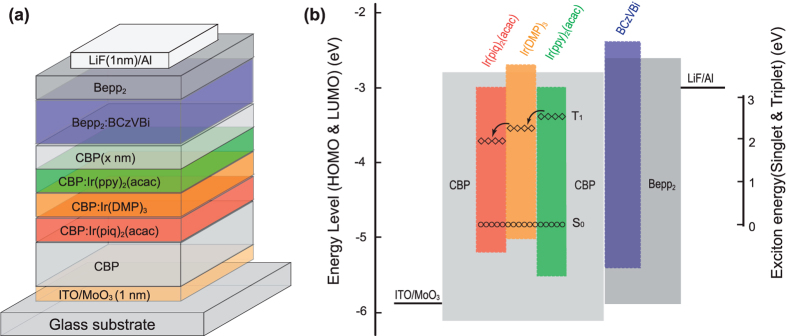
(**a**) Schematic device structure, (**b**) energy-level diagram of the device. The upper and lower boundary of each colored band indicate the LUMO and HOMO position of each molecule used. Circles and diamonds indicate the exciton energies (S_0_ and T_1_).

The optimized structure of the ultrahigh CRI white OLED is ITO/MoO_3_(1 nm)/CBP(20 nm)/CBP: Ir(piq)_2_(acac)(3 wt.%,4 nm)/CBP: Ir(DMP)_3_(5 wt.%,4 nm)/CBP: Ir(ppy)_2_(acac)(7 wt.%,5 nm)/CBP(x nm)/Bepp_2_: BCzVBi(50 wt.%,40 nm)/Bepp_2_(20 nm)/LiF(1 nm)/Al(100 nm). The molecular structures of these organic materials used in this work are shown in Fig. 1. Figure 2(a) shows a schematic device structure and (b) shows the energy-level diagram of the device. By carefully adjusting the doping concentration and thickness of the EML, a full spectrum white light emission can be achieved. Compared to a previous tri-EML white OLED, the four-EML white OLED device shows an ultrahigh CRI of 93.

**Figure 3:**
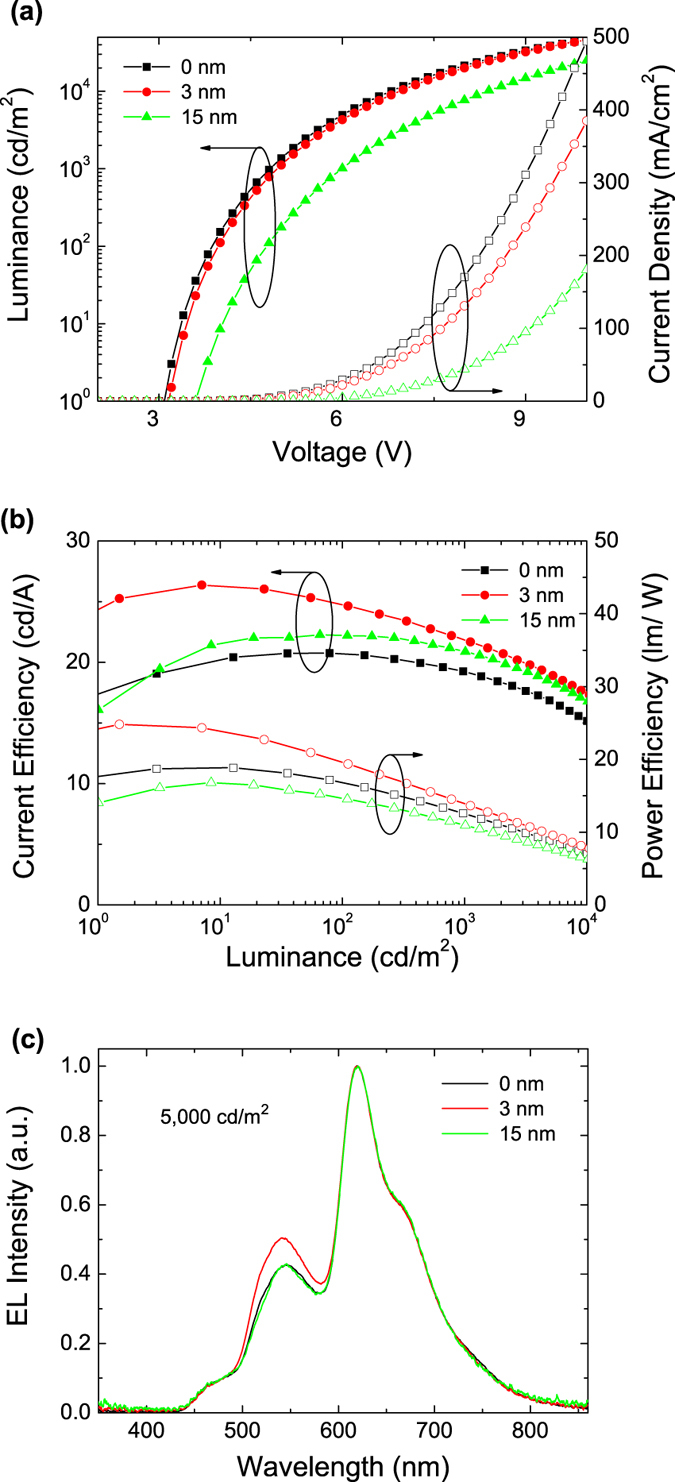
(**a**) L–I–V, (**b**) current and power efficiency-luminance characteristics, (**c**) EL spectra of devices with various thicknesses of spacer layer.

The luminance–current–voltage (*L–I–V*) and the current-power efficiency characteristics of the devices with different thicknesses of CBP spacer layers are shown in Fig. 3. For the optimized device without a spacer layer, it can be seen in Fig. 3a that the luminance and current density increase rapidly with a normal 3.1 V turn-on voltage. This indicates effective injections of both holes and electrons from the electrodes into the device. An ultrahigh CRI of 93, a CCT of 2832 K, a CIE of (0.47, 0.45) at 5000 cd/m^2^, and a power efficiency of 19.1 lm/W are achieved. In order to further increase the efficiencies, we proceed to optimize the thickness of the CBP spacer layer (see Supplementary Fig. S1). As shown in Fig. 3a, the turn-on voltage of the device with CBP layer thickness of 3 nm is almost the same as that of device without a spacer layer, the maximum current and power efficiencies of this device are 26.4 cd/A and 24.8 lm/W respectively without using any out-coupling enhancement techniques. This increase in device efficiency is attributed to a reduced exciton quenching as the spacer layer separates excitons in the blue fluorophore from other triplet emitters. The WOLED with a spacer also yields good lighting parameters including a CRI of 92, a CIE of (0.46, 0.46) and a CCT of 2968 K, at a high luminance of 5000 cd/m^2^. These sets of parameters collectively show this WOLED suitable for a warm-white illumination. These results also indicate that an appropriate CBP spacer layer could increase the current and power efficiencies.

It is worth noting that the current efficiency of the device with the 15 nm spacer is higher than that of device without a spacer layer. This 15 nm spacer layer, however, is larger than the Förster and Dexter energy transfer distance between the blue fluorophore and the phosphors^12^, and thus prevents a direct energy transfer from the blue singlet emitters to the adjacent green triplet emitters. This enhanced current efficiency is probably due to the carrier trapping by the doped triplet emission zones. Charge trapping and energy transfer are two main excitonic processes in doped OLEDs^13^. For the charge trapping process, the dopant molecules will trap the injected charge carriers resulting in a dependence of current density–voltage (*J*–*V)* characteristics on the doping concentration. For the energy transfer process, there are two mechanisms known as Förster energy transfer and Dexter energy transfer. For Förster energy transfer, an exciton on a donor molecule relaxes to the ground state by transferring its energy to an acceptor. For Dexter energy transfer, the donor transfers an unpaired electron from its LUMO to the LUMO of an acceptor, and an electron from the HOMO of the acceptor is simultaneously transferred to the HOMO of the donor. In this energy transfer processes, the dopant molecules function as transition states for the injected unpaired charge carriers, and thus the *J*–*V* characteristics are not influenced by the dopant molecules.

**Figure 4:**
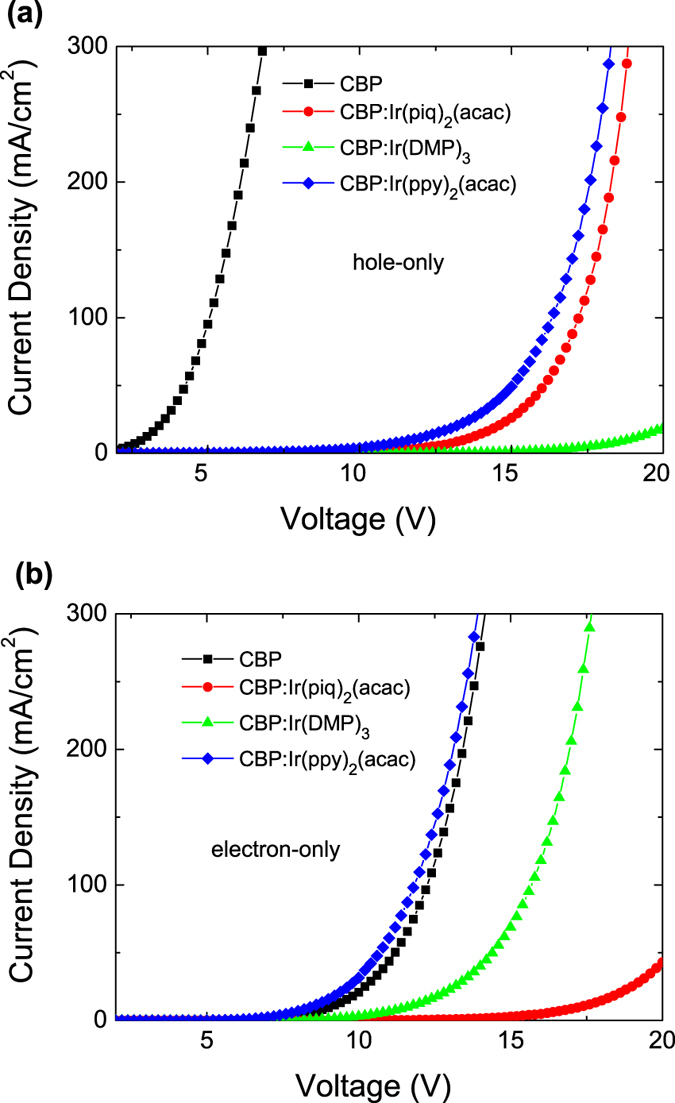
Current density–voltage characteristics of hole-only and electron-only devices. The structure of hole-only device is ITO/MoO_3_(1 nm)/CBP(40 nm)/CBP or CBP: dopant (*x*%,40 nm)/CBP(40 nm)/MoO_3_(10 nm)/Al(100 nm) and the electron-only device is ITO/LiF(1 nm)/Bepp_2_(40 nm)/CBP or CBP: dopant (*x*%, 40 nm)/Bepp_2_(40 nm)/LiF(1 nm)/Al (100 nm).

To elucidate the electroluminescent processes of the WOLED, we investigated *J*–*V* characteristics of hole-only devices [with a structure: ITO/MoO_3_(1 nm)/CBP(40 nm)/CBP or CBP: dopant (*x*%, 40 nm)/CBP(40 nm)/MoO_3_(10 nm)/Al(100 nm)], and electron-only devices [with a structure: ITO/LiF(1 nm)/Bepp_2_(40 nm)/CBP or CBP: dopant (*x*%, 40 nm)/Bepp_2_(40 nm)/LiF(1 nm)/Al (100 nm)]. Fig. 4 shows these single charge carrier *J*–*V* data. It can be seen that the hole current and the electron current are greatly decreased when CBP is doped with Ir(piq)_2_(acac) and Ir(DMP)_3_. This means that Ir(piq)_2_(acac) and Ir(DMP)_3_ play a role of strong hole trapping and electron trapping in the CBP host. Behaved very differently are the Ir(ppy)_2_(acac)-doped CBP devices; the current actually increases in the electron-only device (Fig. 4b) while the hole current decreases in the hole-only device (Fig. 4a). This indicates that Ir(ppy)_2_(acac) traps holes while facilitates electron transport. These characteristics, as will be discussed, are keys for designing emission zones in WOLEDs with low CCT.

The *J*–*V* characteristics of Ir(piq)_2_(acac), Ir(DMP)_3_ and Ir(ppy)_2_(acac) shown in Fig. 4 suggest that these dopants can trap holes in the CBP host. It is known that CBP is an ambipolar host^14^,^15^ and has a peak emission wavelength of λ = 390 nm^16^. This ambipolar spacer layer allows transport of both holes and electrons to EMLs. A lack of CBP emission in these devices suggests that emission from the host is negligible, as is shown in Fig. 3c.

On the basis of the above single charge carrier transport studies, we now discuss the working mechanism of the WOLED. For the electron transport, as described in Fig. 2b, the injected electrons transport through the LUMO level of Bepp_2_ to the LUMO of the spacer CBP layer and to the LUMOs of CBP hosts. As shown above that the green dopant Ir(ppy)_2_(acac) facilitates the electron transport in CBP, so the electrons can be easily transported into the yellow and red emission layer. The flow of electrons, however, will be slowed down in the yellow and red emission layers due to a strong electron trapping by Ir(DMP)_3_ and Ir(piq)_2_(acac) dopant molecules. For the hole transport, the injected holes transport through the HOMO of CBP to the HOMO of the spacer CBP and then to the HOMO of the Bepp_2_. The blue emission is attributed to the energy transfer from the Bepp_2_ to BCzVBi^17^. As discussed above that Ir(piq)_2_(acac), Ir(DMP)_3_ and Ir(ppy)_2_(acac) dopant molecules are capable of trapping holes so direct exciton formation on these emitters plays a major role in these WOLED devices. Additionally, because of the low-lying triplet state of Ir(DMP)_3_ and Ir(piq)_2_(acac), the cascade energy transfer from the green layer to the yellow and red layers also contribute to the yellow and red emission.

**Figure 5:**
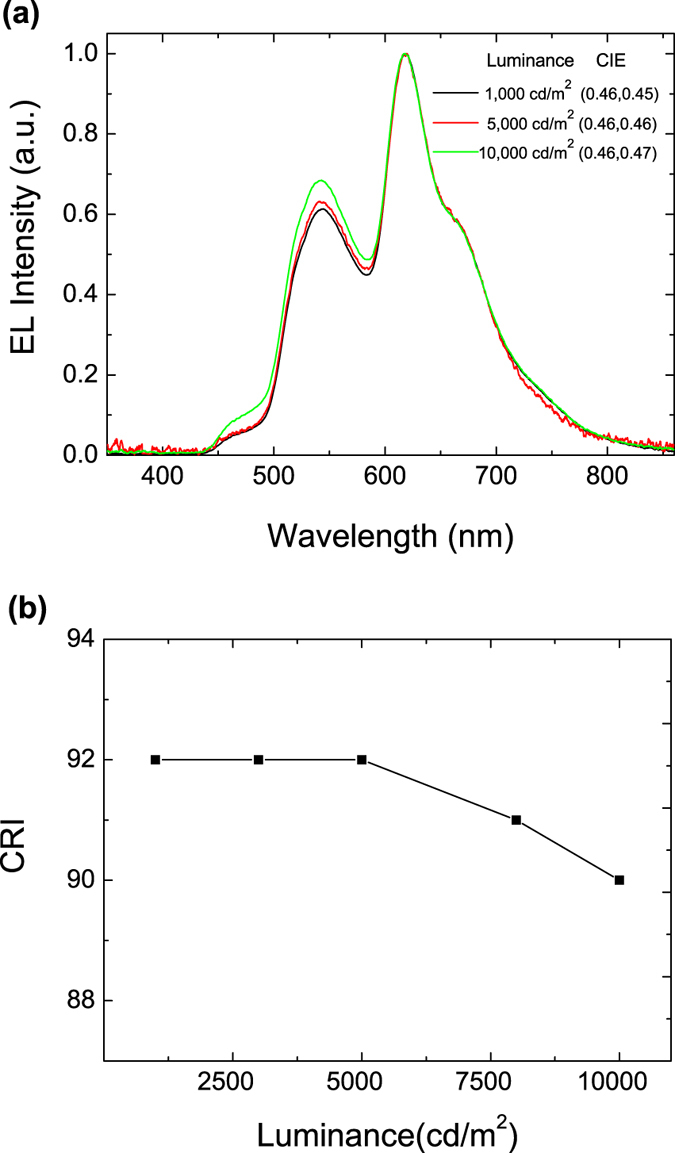
(**a**) EL spectra at different luminance of device ITO/MoO_3_/CBP/CBP: Ir(piq)_2_(acac)/CBP: Ir(DMP)_3_/CBP: Ir(ppy)_2_(acac)/CBP(3 nm)/Bepp_2_: BCzVBi/Bepp_2_/LiF/Al. (**b**) CRI as a function of luminance from 1000 cd/m^2 ^to 10000 cd/m^2^.

Figure 5a shows the EL spectra at different luminance levels for the device of ITO/MoO_3_(1 nm)/CBP(20 nm)/CBP: Ir(piq)_2_(acac)(3 wt.%,4 nm)/CBP: Ir(DMP)_3_(5 wt.%,4 nm)/CBP: Ir(ppy)_2_(acac)(7 wt.%,5 nm)/CBP(3 nm)/Bepp_2_: BCzVBi(50 wt.%,40 nm)/Bepp_2_(20 nm)/LiF(1 nm)/Al(100 nm). The relative intensities of the blue/green to the red emission increase with increasing voltage. This shift shows a significant increase of exciton population taking place in the blue and green emission zones at high voltages. At high applied voltage, the effect of hole trapping is close to be saturated by Ir(piq)_2_(acac) dopants. This leads to relatively more holes being injected into the yellow, the green and the blue emission zones, resulting in an EL enhancement from these emission zones. Despite this shift in exciton distribution, the CRI remains at above 90 at extremely high luminance levels, as is shown in Fig. 5b.

**Figure 6:**
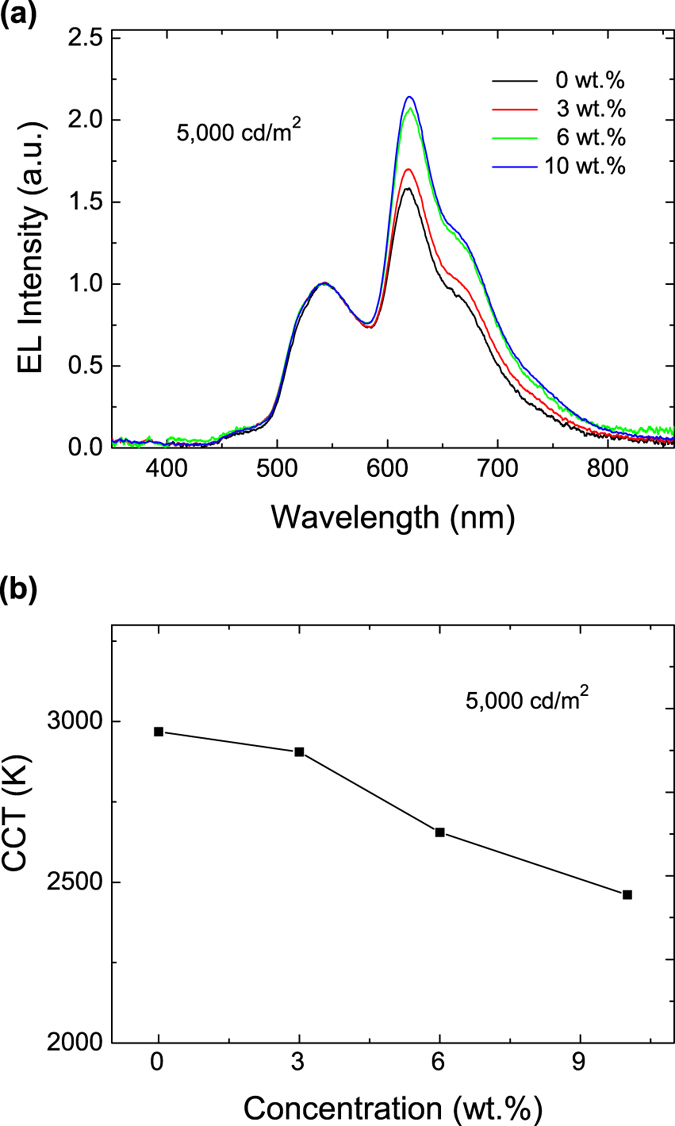
(**a**) The EL spectra of devices with various Ir(ppy)_2_(acac) doping concentration normalized to the green emission peak at 540 nm. (**b**) The CCT as a function of Ir(ppy)_2_(acac) doping concentration at 5000 cd/m^2^.

Now let’s focus on a device engineering method to reduce CCT, which is also a crucial parameter for WOLEDs. Partially based on above experimental findings and partially based on the fact that there is an efficient energy transfer between green and red emitters^12^, we doped Ir(ppy)_2_(acac) into the red emissive layer, i.e., using an intra-zone exciton harvesting scheme, to increase the red color component and thus decrease the CCT of the WOLED. The device is structured as: ITO/MoO_3_(1 nm)/CBP(20 nm)/CBP: Ir(piq)_2_(acac): Ir(ppy)_2_(acac)(3 wt.% R: x wt.% G,4 nm)/CBP: Ir(DMP)_3_(5 wt.%,4 nm)/CBP: Ir(ppy)_2_(acac)(7 wt.%,5 nm)/CBP(3 nm)/Bepp_2_: BCzVBi(50 wt.%,40 nm)/Bepp_2_(20 nm)/LiF(1 nm)/Al(100 m). Figure 6 shows the characteristics of devices with different Ir(ppy)_2_(acac) doping concentrations. Clearly, the green dopant plays an important role in the charge carrier transport and in the harvesting of excitons. As Fig. 4 shows that the Ir(ppy)_2_(acac) traps holes but helps the electron transport, this Ir(piq)_2_(acac): Ir(ppy)_2_(acac) co-doping leads to more excitons formation in the red EML zone. In this intra-zone exciton harvesting system, the fraction of excitons that flows to the Ir(piq)_2_(acac) molecules in the red emission layer can be expressed as^11^:


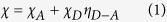


where 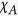 and 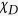 denote the fractions of excitons that formed in the Ir(piq)_2_(acac) (A) and Ir(ppy)_2_(acac) (D) molecules. 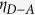 stands for the energy transfer efficiency from green Ir(ppy)_2_(acac) molecules to red Ir(piq)_2_(acac) molecules. According to equation (1), we know that the 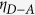 is very high^11^. The green Ir(ppy)_2_(acac) dopants help funnel more excitons into the red Ir(piq)_2_(acac) emitters resulting in an enhanced emission from the red emitter. Fig. 6a shows the EL spectra of device with different Ir(ppy)_2_(acac) doping concentration at a luminance of 5000 cd/m^2^. In addition, the red emission intensity is found to increase with increasing Ir(ppy)_2_(acac) doping concentration. This demonstrates that there are more excitons formed and harvested in the red EML. Shown in Fig. 6b is the CCT as a function of Ir(ppy)_2_(acac) doping concentration. The CCT is found to be reduced by increasing the Ir(ppy)_2_(acac) doping concentration, from ~3000 K to ~2500 K. The CRI of the device remains at ~91 (see Supplementary Fig. S2). This clearly demonstrates that the co-doping intra-zone method provides a practical method to tune the CCT without affecting the CRI value.

In conclusion, high-efficiency and ultrahigh CRI WOLEDs have been demonstrated by using four stacked emission layers with a CBP spacer layer between the blue fluorescent emission zone and the phosphorescent emission zones. The optimized WOLED exhibits a high CRI of 92, a CCT of 2968 K and a CIE of (0.46, 0.46) with a maximum power efficiency of 24.8 lm/W. To tune the CCT of the WOLED without sacrificing the CRI, an intra-zone exciton harvesting by incorporating a green triplet Ir(ppy)_2_(acac) into the red emissive layer is demonstrated to be an effective method.

## Methods

### Materials

The molecular structures of the organic materials used in this work are shown in Fig. 1. These materials were purchased from Luminance Technology Corporation and used in as-received form for device fabrication. We used 4,4′-Bis(carbazol-9-yl)biphenyl(CBP) as an efficient hole transport layer (HTL) as well as the host of phosphorescent dopant. This eliminates the energy barrier at the HTL/EML interface^18^. Bis(1-phenylisoquinoline)(acetylacetonate)iridium(III)(Ir(piq)_2_(acac)), Tris[3-(2,6-dimethylphenoxy)-6-phenylpyridazine]iridium(III)(Ir(DMP)_3_) and Bis(2-phenylpyridine)(acetylacetonate)iridium(III)(Ir(ppy)_2_(acac)) were chosen as red, yellow and green phosphorescent dyes. 4,4′-Bis(9-ethyl-3-carbazovinylene)-1,1′-biphenyl(BCzVBi) doped with Bis(2-(2-hydroxyphenyl)-pyridine)beryllium(Bepp_2_) served as the blue fluorescent emissive unit. Bepp_2_ acted as an electron transport layer (ETL). Bi-layer LiF/Al and MoO_3_/ITO were chosen as the cathode and anode, respectively.

### Device Fabrication

The detailed device structure is depicted in Fig. 2. All devices were fabricated in a tri-chamber high-vacuum thermal evaporation system with a base pressure of ~10^−7^ Torr. The deposition rate was 1.0 Å/s for organics and 0.1 Å/s for LiF and MoO_3_. Finally, aluminum (Al) was evaporated on LiF film at a rate of 2 Å/s. The substrates are commercial patterned indium tin oxide (ITO) coated glass with a sheet resistance of 15 Ω/□. Substrates were cleaned with a standard cleaning procedure^19^. All the organic layers were deposited in an organic chamber. The cathode was deposited in a separate metal chamber. The thickness of each layer was measured by a quartz crystal microbalance that had been calibrated using a spectroscopic ellipsometer.

### Device Measurement

The current–voltage (*I*–*V*) characteristics were measured using a HP4140B picoammeter. The luminance–voltage (*L*–*V*) measurements were performed using a Minolta LS–110 Luminance meter. The electroluminescence (EL) spectra were measured using an Ocean Optics USB4000 spectrometer. The CRI and CCT were measured using an UPRtek MK-350 spectrometer. All measurements were carried out in ambient atmosphere at room temperature.

## Additional Information

**How to cite this article**: Zhang, T. *et al*. A multi-zoned white organic light-emitting diode with high CRI and low color temperature. *Sci. Rep.* **6**, 20517; doi: 10.1038/srep20517 (2016).

## Supplementary Material

Supplementary Information
