# Supplementary material for: A multi-zoned white organic light-emitting diode with high CRI and low color temperature
Source: Sci Rep. 2016 Feb 4;6:20517. doi: 10.1038/srep20517 (PMC4740810; doi:10.1038/srep20517)
Supplement: Supplementary Information [file srep20517-s1.pdf]

Supplementary Information

A multi-zoned white organic light-emitting diode with high CRI and low color temperature

Tao Zhang[1,2], *Shou-Jie He*[1,2], *Deng-Ke Wang*[1,2], *Nan Jiang*[1,2], *and Zheng-Hong Lu*[1,2,3*]

[1]*Department of Physics, Yunnan University, Kunming, Yunnan 650091, People's Republic of China*

[2]*Yunnan Key Laboratory for Micro/Nano Materials & Technology, Yunnan University, Kunming, Yunnan 650091, People's Republic of China*

[3]*Department of Materials Science and Engineering, University of Toronto, Toronto, Ontario M5S 3E4, Canada*

[*] Corresponding author: zhenghong.lu@utoronto.ca

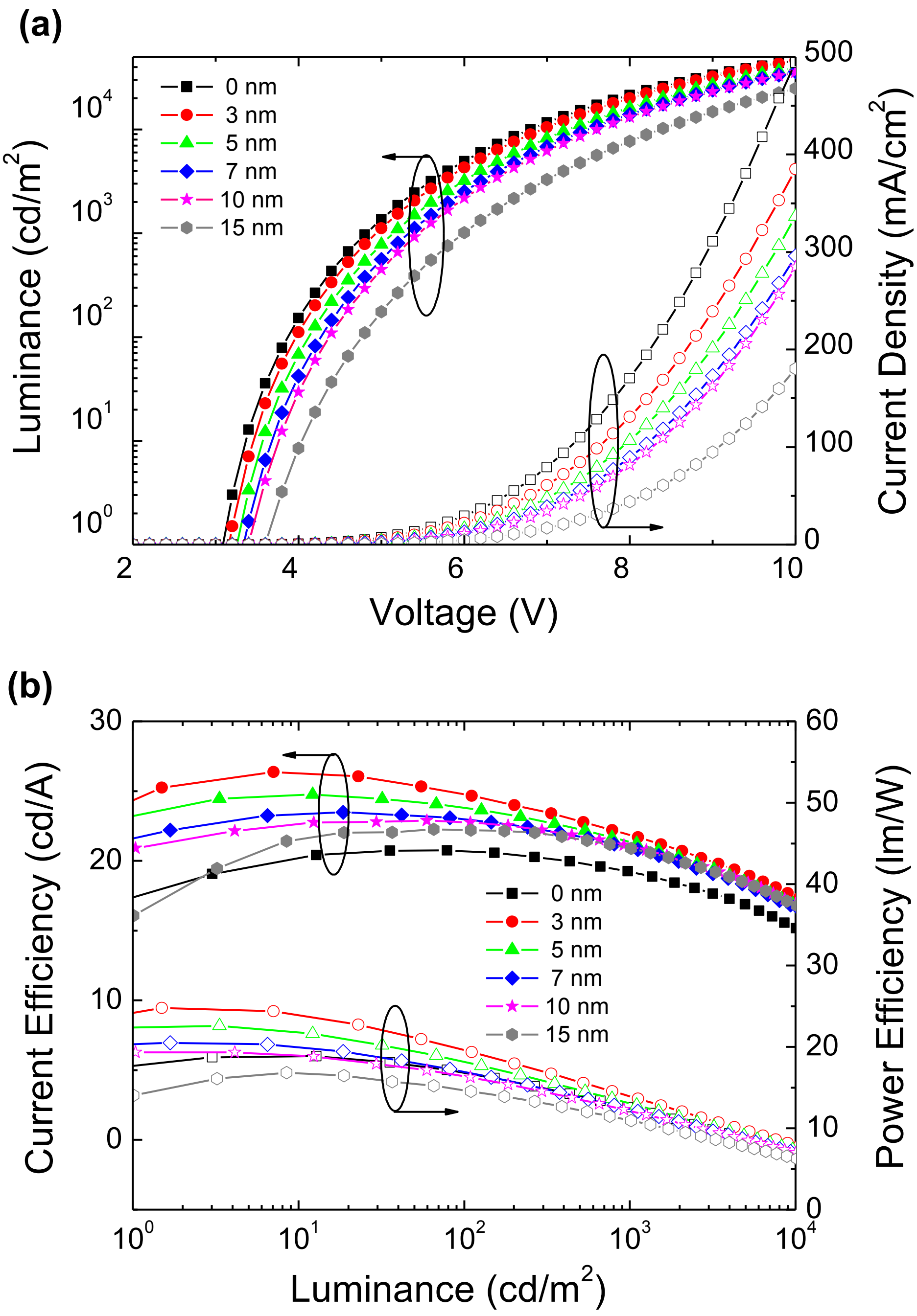

Figure S1 (a) L–I–V, (b) current and power efficiency-luminance characteristics of devices with various thicknesses of the CBP spacer layer.

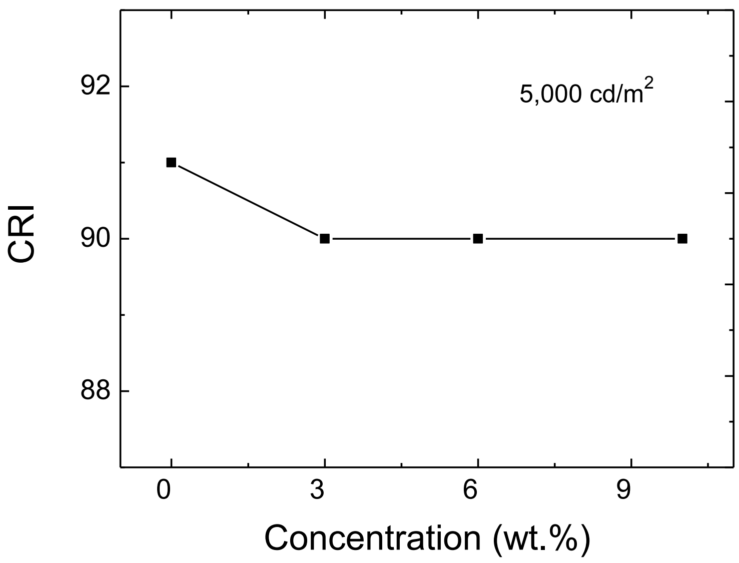

Figure S2 The CRI as a function of $Ir(ppy)_2(acac)$ doping concentration at 5000 cd/m^2.
